# Primary weight maintenance: an observational study exploring candidate variables for intervention

**DOI:** 10.1186/1475-2891-12-97

**Published:** 2013-07-15

**Authors:** Kristina Lindvall, Paul Jenkins, Maria Emmelin, Melissa Scribani, Margareta Norberg, Christel Larsson, Lars Weinehall

**Affiliations:** 1 Department of Public Health and Clinical Medicine, Epidemiology and Global Health, Umeå University, S-901 87 Umeå, Sweden; 2 Umeå Centre for Global Health Research, Department of Public Health and Clinical Medicine, Umeå University, S-901 87 Umeå, Sweden; 3 Bassett Healthcare Network Research Institute, One Atwell Road, Cooperstown, NY 13326 USA; 4 Department of Clinical Sciences, Social Medicine and Global Health, Lund University, Jan Waldenströmsgatan 35, S- 205 02 Malmö, Sweden; 5 Department of Food and Nutrition and Sport Science, University of Gothenburg, Box 300, S-405 30 Gothenburg, Sweden; 6 Department of Food and Nutrition, Umeå University, S-901 87 Umeå, Sweden; 7 Centre for Population Studies, Ageing and Living Conditions Programme, Umeå University, S-901 87 Umeå, Sweden

**Keywords:** Overweight, Obesity, Primary weight maintenance, Obesity prevention, Sweden, Middle-age

## Abstract

**Background:**

Previous studies have focused on weight maintenance following weight loss, i.e. secondary weight maintenance (SWM). The long-term results of SWM have been rather modest and it has been suggested that preventing initial weight gain, i.e. primary weight maintenance (PWM), may be more successful. Therefore, developing a prevention strategy focused on PWM, enabling normal weight or overweight individuals to maintain their weight, would be of great interest. The aim of this study was to identify attitudes, strategies, and behaviors that are predictive of PWM in different age, sex and BMI groups in Northern Sweden.

**Methods:**

A questionnaire was mailed to 3497 individuals in a Swedish population that had two measured weights taken ten years apart, as participants in the Västerbotten Intervention Programme. Subjects were between 41–63 years of age at the time of the survey, had a baseline BMI of 20–30, and a ten year percent change in BMI greater than -3%. The respondents were divided into twelve subgroups based on baseline age (30, 40 and 50), sex and BMI (normal weight and overweight). Analysis of variance (ANOVA), correlation, and linear regression were performed to identify independent predictors of PWM.

**Results:**

Of the 166 predictors tested, 152 (91.6%) were predictive of PWM in at least one subgroup. However, only 7 of these 152 variables (4.6%) were significant in 6 subgroups or more. The number of significant predictors of PWM was higher for male (35.8) than female (27.5) subgroups (p=0.044). There was a tendency (non significant) for normal weight subgroups to have a higher number of predictors (35.3) than overweight subgroups (28.0). Adjusted R-squared values ranged from 0.1 to 0.420.

**Conclusions:**

The large number of PWM predictors identified, and accompanying high R-squared values, provide a promising first step towards the development of PWM interventions. The large disparity in the pattern of significant variables between subgroups suggests that these interventions should be tailored to the person’s demographic (age, sex and BMI). The next steps should be directed towards evaluation of these predictors for causal potential.

## Background

Obesity is the sixth most important risk factor contributing to the worldwide burden of disease [1]. It is associated with cardiovascular disease (CVD), diabetes, cancer, osteoarthritis and chronic pain [1-3] as well as with negative psychosocial and/or psychological effects [4-7]. These negative psychological impacts have been observed in response to negative portrayal in the media, [5] inequities in the employment setting [4,7] and inequities in health care [6].

Many studies have focused on the consequences of being overweight or obese [1-3,8] rather than the relative importance of, and the complex interaction between, factors contributing to these conditions [9,10]. An individual’s weight is the result of a combination of biological, behavioral and environmental factors [11]. Given the current state of scientific research, these behavioral and environmental factors are modifiable by the individual or society, while the biological factors, such as genetic predispositions, are less subject to modification. However, it is important to acknowledge that the effect of a genetic susceptibility to being overweight or obese is also modifiable by behavioral factors such as a physically active lifestyle [12].

Some short-term obesity treatment programs have shown significant weight loss among adults [13,14]. However, this is typically followed by weight gain resulting in rather modest long-term results [15-17]. Further research is therefore needed to understand how successful long-term weight maintenance can be achieved [18]. A shift from focusing exclusively on weight loss, to also including a focus on weight maintenance has been suggested [19-21]. Regardless of the focus, there is no clear consensus on how to intervene to promote either weight loss or weight maintenance [20].

Weight maintenance has been defined as a person's ability to maintain their weight within ± 3% of a baseline value over a defined time period [22]. Previous studies on weight maintenance have most often focused on *secondary weight maintenance* (SWM), i.e. maintaining a reduced weight following weight loss. Two central factors known to be important for SWM are regular physical activity and healthy eating habits [23-27]. Other factors that have been identified include having an accurate self-image [25], a high self-esteem [24], a positive body image [28], consciousness of one’s own behaviour [27], positive self-talk [29] taking responsibility for one’s actions [27], and the ability to cope with stress and confront problems directly [23,27]. Further, successful secondary weight maintainers monitored weight fluctuations and had a clear alarm signal for weight gain that triggered immediate action [30]. They also had clear strategies for coping with lifestyle interruptions. When compared with re-gainers, maintainers more often continued to use the strategies they had acquired during weight loss [29]. In addition, a study on mediators of weight loss and weight loss maintenance showed that lowering emotional eating and adopting a flexible dietary restraint pattern were critical for sustaining weight loss [31].

In several studies on SWM, the subjects did not fare well at maintaining their weight after weight loss [13-17]. It is possible that this limited success is partially a consequence of the antecedent weight gain. It can therefore be theorized that a subject who has not experienced this initial weight gain may be more successful in weight maintenance. We propose that with two subjects with a Body Mass Index (BMI) of 24, the subject who has reduced his/her weight to reach that BMI will have more difficulty remaining there than the subject who never has exceeded a BMI of 24. Further, enabling subjects to prevent initial weight gain spares them the difficulty of trying to get down to a lower weight. Prevention of initial weight gain would also reduce the large burden placed on the entire population to lose weight.

The concept of preventing weight gain among normal weight or overweight individuals is called *primary weight maintenanc*e (PWM).

Qualitative interviews conducted previously by the authors identified four main strategies related to PWM: “to rely on heritage”, “to find the joy”, “to find the routine” and “to be in control” [32]. Nested within these four main strategies were eleven “ideal types”. These ideal types were theoretical constructs that captured the attitudes, strategies and behaviors related to weight maintenance. Knowledge of how these factors are distributed within the population would contribute to a deeper understanding of PWM and lay the ground work for the development of intervention programs.

Since PWM has received relatively little attention in the literature, there are currently no obesity prevention programs that are employing it. While the assumption may exist that an intervention focused on PWM would proceed in an identical manner to one directed at weight loss and/or SWM, this has not been scientifically established. Given the limited long-term success of weight loss programs in general, a new approach, starting with the development of an understanding of the factors related to PWM, is warranted.

### Aim

To identify attitudes, strategies, and behaviors that are predictive of PWM in different age, sex and BMI groups in Northern Sweden.

To quantitatively validate the existence of the eleven ideal types that were previously identified in a qualitative study.

## Methods

### Setting

The study was conducted in Northern Sweden in Västerbotten County in 2009. The population of Västerbotten is approximately 260,000. About 40% live in the largest city with the remainder in two smaller cities or the surrounding countryside.

### Study respondents and the Västerbotten Intervention Programme

The subjects had participated twice in the Västerbotten Intervention Programme (VIP) which was launched in 1985 to reduce risk factors for CVD and diabetes [33]. The VIP was integrated into routine health care delivery, with all inhabitants invited to participate on turning 30, 40, 50 and 60 years of age. The health examinations include assessment of risk factors for CVD and an extensive questionnaire regarding socioeconomic status, marital status, self reported health and lifestyle habits. Consecutive cross-sectional data from the VIP showed that the prevalence of overweight and obesity had increased among men from 47.2% and 10.0% (overweight vs. obese) as of 1995 to 49.4% and 17.3% as of 2007 [34]. For women the prevalence had increased from 32.2% and 12.7% as of 1995 to 33.3% and 16.5% as of 2007.

In order to participate in the present study, the respondent had to be between 41–63 years of age in 2009. Eligible subjects needed a baseline VIP measured weight in 1993–1998, and a second weight taken ten years later. Using the baseline BMI value, two equally wide BMI strata were formed: normal weight (20–25), and overweight (25–30). Subjects were also stratified based on baseline age (30, 40 and 50) and gender. Subjects with a BMI of less than 20 or greater than 30 were excluded. Finally, any subject who had lost more than 3% of their baseline BMI was excluded in order to omit subjects with weight loss secondary to health problems.

### The questionnaire

The questionnaire was constructed based on the results and hypotheses developed from previous quantitative- [35] and qualitative studies of weight maintenance [32]. It also included five questions from the VIP questionnaire and seven others obtained from US collaborators. The questionnaire was piloted among 35 individuals with a similar demographic profile as the study population. The final version included 31 questions (with a number of sub-questions) that assessed attitudes, strategies and behaviors regarding food habits, physical activity, tobacco use, body image and demands to maintain weight. Some groups of questions could also be linked to the “ideal types” developed previously [32] (see Additional file 1). For example, the statement: *“I eat food that is nutritious regardless of taste”* was related to the “The health concerned eater” ideal type.

The first mail-out, in late May 2009, included a questionnaire and information about the study. Because of a mailing delay, a reminder post card was sent approximately one week later informing the subjects that, even if the stated deadline for returning the survey had passed, they should still complete and return it. Those that had not completed the survey by mid June received a third mailing containing a new questionnaire and a renewed request to complete it. The subjects did not receive any incentives for participating.

### Response rate

In total, 3497 individuals were selected for the study. Ten of these did not receive questionnaires due to a mailing error or invalid address. Of the remaining 3487, 1893 agreed to participate giving a response rate of 54.3%. Eighty-nine subjects were excluded because they did not give consent for linkage to the VIP database. Finally, three individuals were excluded because their ten-year VIP follow-up date was more than five years before the administration of the survey. Thus, usable data were available for 1801 individuals.

### Data analysis

PWM was operationally defined as 10-year percent weight change calculated as:

$$\frac{\begin{array}{l} \;\text{The measured weight at the second VIP visit}-\\ \;\text{The measured weight at the first VIP visit} \end{array}}{\begin{array}{c} \text{The measured weight at the first VIP visit} \end{array}}\;\times 100$$

The design of the study called for 150 respondents in each of the 12 age, sex and BMI strata. With this sample size, power to detect differences of 7.5 percent weight change across levels of the Likert scale responses, using a one-way Analysis of variance (ANOVA), was estimated to be 81.2%.

To minimize sparseness, five-level Likert scale variables were collapsed into three levels. Thus, “strongly agree” and “agree” were reclassified into “agree”, “unsure” remained as “unsure”, and “disagree” and “strongly disagree” were reclassified into “disagree”. Likewise, “always” and “often” became “usually”, “sometimes” remained as “sometimes”, and “seldom” and “never” were reclassified as “rarely”. In certain limited cases, where sparseness remained, a combination of visual inspection and statistical analyses were used to further combine levels. Questions where one response level (for example “agree”) was selected by more than 96% of the respondents were eliminated from further consideration. Finally, there were two questions where the subject indicated their body size, at both baseline and ten-year follow-up, using a nine point scale. Responses from women who stated that they were pregnant at either of these two time points were set equal to missing.

### Item analysis and factor analysis

One goal of the study was to reduce the number of variables under consideration. Two separate avenues were pursued to accomplish this. First, the correlation between survey variables was examined with the intention of eliminating one member of any pair with a correlation between them of 0.95 or higher. This did not lead to the elimination of a single variable.

The second analytic attempt at dimension reduction employed factor analysis. Specifically, a confirmatory factor analysis to identify the eleven ideal types was performed using principal factor extraction and varimax rotation of basis vectors. These analyses were conducted separately for men and women. Neither the scree plot of the Eigenvalues, nor the pattern of factor loadings, indicated the presence of these eleven ideal types. A second analysis based on an a-priori identification of six factors also did not produce a satisfactory mathematical solution. Therefore, rather than using eleven or six principle components in subsequent analyses, the study retained the original set of 166 survey variables for use in the prediction of PWM.

### Prediction of PWM

Four analytic steps were conducted to identify variables that were predictive of PWM. First, a one by three ANOVA was used within each of the twelve subgroups to compare mean percent weight change across the three levels of the collapsed Likert scale responses. In certain limited cases where further collapsing into only two levels was required, this resulted in a t-test. In addition, Pearson correlations between these three response levels and percent weight change were calculated. If the significance test for either the ANOVA or the correlation was significant at p ≤ .10 the variable was retained for the next step of the analyses. The number of significant variables identified by these analyses was summarized for each subgroup in tabular form.

At this point, an attempt was made to combine subgroups together based on a similar pattern of significant predictors of PWM. This involved the creation of frequency tables that showed how many of the twelve subgroups (six for each sex) the variable was significant in. Further, the subgroups were examined visually to attempt to identify those that had the same set of significant predictors. For reasons that will be clarified in the results section, it was immediately apparent upon visual inspection that combining subgroups was not appropriate. This required that the analyses continue stratified by all twelve groups.

Following this attempt to combine subgroups, step two was undertaken to identify independent predictors of PWM. Taking variables with p ≤ .10 at the univariate level, a stepwise linear regression model was used with a significance level to both enter and remain in the equation set at p ≤ .05. This resulted in a final reduced set of variables for the prediction of PWM within each of the twelve subgroups.

After completion of the stepwise regressions, the models were rerun using only the final set of independent predictors. This was done to retain as many respondents as possible, as this final regression excluded only respondents with missing data on the final set of predictors, whereas the stepwise models excluded respondents missing any of the larger set of original candidate variables. Each of the twelve groups was then summarized as to the number of independently significant predictors and the proportion of variance explained (R-squared).

The regression models described above (step 2 and 3) were also run on a reduced set of variables that were deemed by the research group to be subject to modification by the individual or society (referred to here as modifiable variables).

The number of significant predictors and the proportion of variance explained (R-squared) were contrasted between men and women and between normal versus overweight groups. For the male versus female comparison, matched pairs were formed, by comparing the appropriate age and BMI group between the two sexes. For example, the normal weight 30 year old female value formed a matched pair with the normal weight 30 year old male value. Similar logic was applied to the comparison of the two BMI strata. In each case this resulted in an analysis of six matched pairs using a dependent samples t-test. These analyses were performed both for predictors significant at the univariate level and also for the subset of independently significant predictors.

Both the number and proportion of significant variables representing each ideal type were summarized in table form. The proportions were calculated within the subgroups by dividing the number of significant variables related to each ideal type by the total number of variables representing it.

### Ethical considerations

This study was approved by the regional Research Ethics Board in Umeå (Dnr 06-071M). Individuals gave informed consent prior to each health screening and also when filling in the questionnaire. They were given three alternatives; to not participate, to participate but not allowing any linking to the VIP database and to participate and allow linking to VIP-data.

## Results

Responders were slightly older (53.3 years) than non-responders (51.6 years), p<0.001 and had a slightly lower percent weight change (4.8 versus 5.9%), p<0.001. Responders also had a lower baseline BMI (24.7 kg/m^2^ versus 25.0 kg/m^2^), p<0.001. Those with a higher education were also more likely to respond (responders: basic 11.4%, mid-level 59.3%, and high 29.3%), (non-responders: basic 13.6%, mid-level 62.2%, and high 24.2%), p<0.001.

Among the 1801 respondents with usable data, 52.9% were women and 47.1% were men. The mean age for women and men were 53.1 (SD=7.0) and 53.5 years (SD=7.3) respectively. The number of respondents in each subgroup ranged from 112 to 188 (Table 1).

**Table 1:** Number of variables significantly related to PWM within each baseline subgroup

**Weight category**	**Normal weight**			**Overweight**			**All**
**Age group (y)**	**30**	**40**	**50**	**30**	**40**	**50**	
*Women*							Average
Respondents (no. and (%) of total)	149	190	188	114	156	156	
	(8.3)	(10.5)	(10.4)	(6.3)	(8.7)	(8.7)	
No. of significant variables	21	33	35	31	17	28	27.5
No. of independently significant variables	4	3	3	7	2	4	3.83
*Men*							
Respondents (no. and (%) of total)	120	142	172	112	132	170	
	(6.7)	(7.9)	(9.6)	(6.2)	(7.3)	(9.4)	
No. of significant variables	34	45	44	24	29	39	35.83
No. of independently significant variables	4	8	6	4	5	7	5.67

### Item analysis and checks for redundancy

For the 166 variables considered, there was no correlation between any two higher than 0.64 for women and 0.56 for men. Therefore, as stated above, this analysis did not lead to the elimination of any variables.

### Factor analysis

For the factor analysis that sought to confirm the presence of the eleven ideal types, the scree plot of the Eigenvalues did not show a noticeable break at the eleventh value for either males or females. The proportion of total variance explained by the first eleven factors was 41.9% and 41.2% for men and women respectively. There was no discernible pattern in the factor loadings that suggested the presence of the eleven ideal types.

Results for the six-factor solution were equally unsatisfactory. For both genders, less than 25% (22.8% for men, 21.7% for women) of the variance was explained for either six-factor solution. Neither the scree plot of the Eigenvalues nor the pattern of factor loadings gave support to the presence of six factors to explain the variance in the system of variables.

### Patterns for individual variables

Variables that were significantly predictive of PWM differed greatly between subgroups in both number and type. This was true both for the entire predictor set, and also for the subset of independent predictors.

The mean number of significant predictors was higher for the six male (35.8) than the six female (27.5) subgroups (p=0.044) (Table 1). The six normal weight subgroups (35.3) also tended to have a higher mean number of predictors than the six overweight subgroups (28.0), however this difference did not reach statistical significance (being based on only six matched pairs of subgroups, these analyses had only limited statistical power). These same general patterns were observed when considering only the subset of modifiable variables (data not shown).

When comparing only independent predictors between the demographic subgroups, the results were less striking than those seen for the entire predictor set (Table 1). Specifically, while the average number of independent predictors was again higher for men (mean 5.7) than women (mean 3.8) this difference did not reach statistical significance. Further, the average numbers of independent predictors for normal weight versus overweight subjects were virtually identical (mean 4.7 vs. mean 4.8).

### Patterns for ideal types

Within subgroups, the significant predictors of PWM represented an average of 6.3 of the 11 ideal types (Table 2). Typically, for each ideal type that was represented, only a small proportion of the total number of the variables that were attached to it was significant (shown in parenthesis in Table 2). In only four of the twelve subgroups did any of these proportions exceed ≥0.5. Thus, not only were the significant predictors for each subgroup dispersed over a large number of ideal types, but within a given ideal type, only a small subset of the variables comprising it were predictive.

**Table 2:** Number and proportion of variables assigned to each ideal type found to be significantly related to PWM

**Ideal type (Number of variables related to the ideal type)**	**Number of variables significantly related to ideal type (Proportion**^**a**^**)**					
	**Normal weight**			**Overweight**		
**Age group (y)**	**30**	**40**	**50**	**30**	**40**	**50**
*Women*						
The unstructured eater (7)	2 (0.29)	0	0	2 (0.29)	0	1 (0.14)
The habitual eater (7)	0	0	0	1 (0.14)	0	1 (0.14)
The holistic eater (7)	2 (0.29)	3 (0.43)	1 (0.14)	1 (0.14)	0	0
The family pleaser (8)	0	0	3 (0.38)	0	0	0
The weekend celebrator (6)	0	0	1 (0.17)	1 (0.17)	1 (0.17)	2 (0.33)
The health concerned eater (7)	2 (0.29)	**4 (0.57)**	1 (0.14)	0	1 (0.14)	2 (0.29)
The enjoyment seeker (3)	0	1 (0.33)	0	1 (0.33)	0	1 (0.33)
The competing athlete (5)	1 (0.2)	1 (0.2)	0	2 (0.4)	1 (0.2)	0
The daily exerciser (6)	0	**3 (0.5)**	**3 (0.5)**	1 (0.17)	0	2 (0.33)
The emotion releaser (3)	1 (0.33)	0	1 (0.33)	0	0	0
The health concerned exerciser (6)	1 (0.17)	0	1 (0.17)	0	0	0
*Men*						
The unstructured eater (7)	2 (0.29)	1 (0.14)	1 (0.14)	0	2 (0.29)	2 (0.29)
The habitual eater (7)	3 (0.43)	0	1 (0.14)	1 (0.14)	0	2 (0.29)
The holistic eater (7)	2 (0.29)	3 (0.43)	0	3 (0.43)	2 (0.29)	1 (0.14)
The family pleaser (8)	1 (0.13)	1 (0.13)	2 (0.25)	1 (0.13)	0	2 (0.25)
The weekend celebrator (6)	1 (0.17)	0	1 (0.17)	1 (0.17)	1 (0.17)	2 (0.33)
The health concerned eater (7)	1 (0.14)	3 (0.43)	0	0	1 (0.14)	0
The enjoyment seeker (3)	0	0	1 (0.33)	0	1 (0.33)	0
The competing athlete (5)	0	0	0	0	2 (0.4)	2 (0.4)
The daily exerciser (6)	0	1 (0.17)	0	0	0	1 (0.17)
The emotion releaser (3)	1 (0.33)	1 (0.33)	**2 (0.67)**	0	0	0
The health concerned exerciser (6)	2 (0.33)	**3 (0.5)**	**3 (0.5)**	1 (0.17)	1 (0.17)	0

^a^ Proportion of significant predictors representing an ideal type/ total number of predictors representing that ideal type. Proportions ≥0.5 are marked with bold text.

### Results for R-squared

The adjusted R-squared values from the linear regressions quantify the amount of variability in PWM that is explained by a linear composite of predictors. These values ranged from 0.1 to 0.42 across the twelve subgroups (Table 3). Female subgroups had lower average R-squared values than male subgroups (0.22 vs. 0.33). There were also lower average R-squared values for the overweight compared to the normal weight subgroups (0.26 vs. 0.29). There was not a monotonic trend in R-squared values with respect to age, with values of 0.29, 0.20 and 0.33 in the 30, 40 and 50 year old groups respectively.

**Table 3:** Proportion of variance explained using both modifiable and non modifiable variables

	**Normal weight**			**Overweight**		
**Age group (yrs)**	**30**	**40**	**50**	**30**	**40**	**50**
*Women*						
(No of respondents)	149	190	188	114	156	156
R-squared all variables	0.191	0.100	0.322	0.379	0.140	0.248
R-squared modifiable variables only	0.204	0.137	0.308	0.291	0.108	0.321
*Men*						
(No of respondents)	120	142	172	112	132	170
R-squared all variables	0.358	0.321	0.420	0.239	0.218	0.393
R-squared modifiable variables only	0.384	0.276	0.275	0.066	0.171	0.382

### Modifiable predictors

Table 4 shows the lists of modifiable variables that were independently predictive of PWM for the three subgroups with the highest r-squared values. Some of the variables shown in the table are statements such as “I am active when I have friends to accompany me”. A plus sign following these statements indicates that respondents who agreed with them tended to gain more weight, while a negative sign indicates the opposite. Other variables are characteristics of the respondent, such as the highest level of education completed, for which a plus sign indicates that the higher the level the greater the weight gain. A third set of variables relates to attitudes of the respondent such as “The image that is most compatible with the way I would like to look has number (1–9)”. This question has a companion question that asks “The image that best describes how I look today has number (1–9)”. For both of these questions, a higher number indicates a larger body size. Therefore, the negative sign following these two questions indicates that the larger the body size selected by the subject, the smaller the gain in weight. Finally, the table contains a variable that represents the difference between how the subject would like to look minus how he actually does look. Thus, individuals who would like to be thinner than they actually are would be assigned negative values on this difference score and vice versa. Therefore, when the question representing this difference score is followed by a plus sign, this indicates that individuals desiring to be thinner than they actually are tend to have lower percent weight change.

**Table 4:** List of modifiable variables found to be independently related to PWM within three subgroups with the highest R-squared values

**Women 50 overweight**	**Men 30 normal weight**	**Men 50 overweight**
**(R-square= 0.32)**	**(R-square=0.38)**	**(R-square=0.38)**
**Significant variable (sign of correlation)**	**Significant variable (sign of correlation)**	**Significant variable (sign of correlation)**
I do not eat the way I wish to do because I am ill (+)	Highest completed level of school (+)	I eat food that suits my own needs (-)
I am active when I have friends to accompany me (+)	I eat in different ways on the weekdays (-)	I eat “healthy” on the weekdays and more “unhealthy” on the weekend (-)
I am physically active to accomplish work or transportation (+)	I eat about the same amount of food every day (-)	I have forbidden myself from eating (certain) unhealthy foods (+)
Food affects my health (-)	I finish my portion when eating out (-)	I think it is fun to test my limits through physical activity (-)
Difference between how I wish to look and how I look (+)	I do not eat the way I wish to do because I am too tired (+)	The image that is most compatible with the way I would like to look has nr (-)
I eat different food during different seasons (-)	Difference between how I wish to look and how I look (+)	Difference between how I wish to look and how I look (+)

### Patterns for variables significant in half or more of the subgroups

Table 5 tabulates how many subgroups each of the 166 variables was significant in for each sex. As shown, of the variables that were significant, the vast majority (90.4% for women and 87.3% for men) were significant in less than half of the subgroups. Taken across both sexes combined, of the 152 variables that were significant in at least one subgroup, only 7 of these (4.6%) were significant in 6 subgroups or more (data not shown). As may be surmised, as a consequence of this, the variables that were significantly predictive of weight change differed widely between groups with no two groups having the same set of predictors. It was this result that led the investigators to conclude that combining either BMI or age subgroups would obscure this important aspect of the data.

**Table 5:** Gender-specific distribution of the number of subgroups that a variable is significant in

**Number of subgroups for which variable is significant**	**Frequency**	**Percent**
*Women*		
0	63	38
1	60	36.1
2	27	16.3
3	13	7.8
5	3	1.8
6	0	0
*Men*		
0	47	28.3
1	57	34.3
2	41	24.7
3	16	9.6
5	2	1.2
6	3	1.8

## Discussion

The high R-squared values indicate that the questions developed from the qualitative study [32], were of relevance in explaining PWM. However, the manifestation of these variables was contrary to what was expected. More specifically, it was anticipated that if one variable from an ideal type was a significant predictor within a subgroup, then most or all of the other questions comprising that ideal type would also be significant. This hypothesis was not borne out by the results. In fact, it was typical for a limited subset of predictors from several different ideal types to form the predictor set within a given subgroup. It was rare for any ideal type to have even half of its component questions be significant within a subgroup. From this, it must be concluded that it would not be possible to classify the mechanisms of PWM within a subgroup using these ideal types [32]. This conclusion was also supported by the results of the factor analyses.

The findings of a higher number of significant predictors and higher R-squared values for men may suggest that weight maintenance may be a more complex issue for women than men. Among the issues faced by women are having lower caloric requirements than men yet being served the same portion sizes by restaurants [36]. Women also report a greater tendency to overeat during stress [37]. Further, care giving responsibilities and safety concerns can place limits on the time and places where women can obtain exercise [38,39]. For some ethnic groups, there may also be fewer acceptable forms of physical activity for women than men [38].

In addition to the pattern of significant predictors representing a wide dispersion of ideal types, it was also observed that there was a great disparity in these predictors between subgroups. This implies that a future intervention has to be tailored based on age, sex and BMI. This approach has been suggested in an article summarizing lifestyle recommendations to prevent weight gain and achieve weight loss among children and teenagers [40]. It has also been suggested that this tailored approach would be beneficial for the treatment of obesity and SWM [41]. Further, for reasons explained above, an intervention could not be centered on the concept of an ideal type.

When designing an intervention, it must be born in mind that this study has only identified variables that are related to PWM, and has not established any causal connections, which can only be done in a randomized intervention. It is also important to note that a study of this type is vulnerable to the issue of reverse causality. Thus, when two variables have a causal connection, the issue of which was the cause of the other could also only be clarified in a prospective trial. Finally, even if causal connections were established, a variable would only be useful in an intervention if it was something that the individual or society could control.

Before planning an intervention, there are several decisions an investigator would be faced with, the initial being which subgroups to intervene on. One logical approach would be to begin with those subgroups where the greatest degree of the phenomena is explained by modifiable variables (the age/sex- and BMI-strata with the highest R-squared) (Table 4). The significant predictors of PWM within these subgroups would then be evaluated for their potential for having causal effects.

### Methodological considerations

Due to a mailing delay, the subjects did not receive the questionnaire that was sent initially until after the stated deadline to return it. While it is known that certain subjects did not return the questionnaire because of this, the exact effect on the response rate cannot be quantified.

The use of an arbitrary cut off to define PWM was avoided for several reasons. First, this convention would place limitations on both the analytic possibilities and statistical power of the study. Second, and more importantly, a change in weight over a ten year period is an outcome that exists on a continuum. Creation of arbitrary cut points for who has maintained versus who has not maintained weight invariably creates situations in which individuals with widely disparate patterns of weight change are placed in the same group. Conversely, individuals for whom the difference in weight gain is trivial may be placed in different groups if these weight changes are near the cut point. Thus, while it is tempting to want to classify individuals as having maintained versus having gained weight, this arbitrary classification has little actual connection to the continuum of weight change and is not conducive to exploring the phenomenon.

Due to the large number of statistical tests, the experiment-wide alpha is very high. Given this, it is almost certain that some of the significant findings represent type 1 errors. Despite this, a correction, such as Bon Ferroni was not employed in order to maximize the number of variables that would be identified for their potential value in an intervention.

The vast majority of respondents (close to 80%) reported that the change in their weight over the ten-year period was essentially monotonic i.e. generally trending consistently up, consistently down, or remaining the same. Thus, while some respondents’ perspectives were reflective of episodes of SWM, it is believed that the vast majority of responses are reflective of PWM.

## Conclusions

The large number of predictors of PWM that were identified, and accompanying high R-squared values, provide a promising first step towards the development of PWM interventions. The widely disparate pattern of predictors in the twelve subgroups suggests that future interventions would need to be tailored based on a population’s demographic (age, sex and BMI). Further, the predictors that were identified require assessment with regard to their potential for causation. Historically, attempts to stem the global obesity epidemic have focused primarily on SWM. This study proposes an alternative focus on PWM, centered on reducing the population’s need to lose weight by seeking to prevent initial weight gain.

Further study should be directed at developing a formal process to involve experts in variable selection for interventional trials.

## Abbreviations

SWM: Secondary weight maintenance; PWM: Primary weight maintenance; VIP: Västerbotten intervention programme; BMI: Body mass index; ANOVA: Analysis of variance.

## Competing interests

The authors declare that they have no competing interests.

## Authors’ contributions

KL constructed the questionnaire, led the implementation of the study, performed all data analyses, and drafted most of the manuscript. PJ guided the construction of the questionnaire, guided the data analyses, helped in interpreting the findings and drafted parts of the method, results and discussion section. ME guided in the construction of the questionnaire, helped to interpret the findings and drafted parts of the discussion sections. MS provided critical revisions. MN provided conceptual guidance and critical revisions. CL guided in the construction of the questionnaire, helped to interpret the findings, drafted parts of background and the discussion. LW guided the construction of the questionnaire, contributed to study design and the implementation of the study, helped to interpret the findings and drafted parts of the discussion. All authors edited the entire manuscript, and read, revised and approved the final version.

## Supplementary Material

Additional file 1 Main strategies and ideal types from a qualitative sub-study and the variables derived from that sub-study that were later used in the questionnaire. Click here for file
