# Supplementary material for: Primary weight maintenance: an observational study exploring candidate variables for intervention
Source: Nutr J. 2013 Jul 15;12:97. doi: 10.1186/1475-2891-12-97 (PMC3717287; doi:10.1186/1475-2891-12-97)
Supplement: Additional file 1 — Main strategies and ideal types from a qualitative sub-study and the variables derived from that sub-study that were later used in the questionnaire. [file 1475-2891-12-97-S1.doc]

**Appendix 1. Main strategies and ideal types from a qualitative sub-study and the variables derived from that sub-study that were later used in the questionnaire.**

| **Main strategy used to maintain weight** | **Ideal type (representing attitudes, strategies and behaviours contributing to weight maintenance** | **Characterized by these questions in the questionnaire:** |
| --- | --- | --- |
| **To rely on heritage** | **The habitual eater** | 1. I most and foremost eat food that keeps my blood sugar under control 2. I eat about the same amount of food every day 3. I eat at the same times every day 4. I eat the same regardless if it is a weekend or a weekday 5. I eat similar foods to those that I ate as a child 6. I reward myself with things other than food 7. Food holds no great importance for me. It is more of a routine |
|  | **The unstructured eater** | 1. I most and foremost eat food that suits my own needs 2. I eat about the same amount of food every day (I do not) 3. I eat at the same times every day (I do not) 4. I eat without taking much notice of it 5. I eat almost anything 6. I only eat according to my own needs 7. Food is fuel that gives me the energy to do what I need to do |
| **To find the joy** | **The holistic eater** | 1. I most and foremost eat food that is nutritious and taste good 2. I limit my alcohol intake 3. I eat locally grown produce 4. I eat organic foods 5. I only eat food that is well-prepared/cooked 6. I choose food that gives pleasure and delight 7. Food gives me a sense of well-being |
|  | **The competing athlete** | 1. I am most and foremost physically active to compete with myself and/or others 2. I maintain my physical activity habits even during vacations 3. I meet new friends through physical activity 4. I establish goals for my training 5. I think that it is fun to test my limits through physical activity |
|  | **The enjoyment seeker** | 1. I am most and foremost physically active for fun 2. I am active when I have friends to accompany me 3. I reward myself by being physically active |
| **To find the routine** | **The weekend celebrator** | 1. I most and foremost eat food that helps me maintain my weight 2. I eat “healthy” on weekdays and more “unhealthy” on the weekend 3. I eat more calories on the weekends and fewer calories on the weekdays 4. I have forbidden myself from eating certain unhealthy foods 5. I reward myself with food 6. Healthy food is boring, less healthy food is exciting |
| **Main strategy used to maintain weight** | **Ideal type (representing attitudes, strategies and behaviors contributing to weight maintenance** | **Characterized by these questions in the questionnaire:** |
| **To find the routine**  **cont.** | **The daily exerciser** | 1. I am most and foremost physically active to maintain energy levels 2. I am most and foremost physically active to accomplish work or transportation 3. I exercise weekdays/daily 4. I do heavy physical labor 5. I exercise close to home 6. I exercise regularly but not if it is in conflict with family time |
|  | **The family pleaser** | 1. I most and foremost eat food that suit both me and my family/others close to me 2. I limit the portion size of what I eat 3. I eat similar foods to those that I ate as a child but choose the "lite" alternative today 4. I eat food that can help me avoid gaining weight 5. I plan my weekly meals 6. My food choices suit both myself and other household members 7. I want to be a role model for my children and/or other close relations by eating healthy 8. Food is a social activity |
| **To be in control** | **The emotion releaser** | 1. I am most and foremost physically active to maintain my weight 2. I exercise to decrease anxiety and anger levels 3. I exercise to release tension and clear my mind |
|  | **The health concerned eater** | 1. I most and foremost eat food that is nutritious regardless of taste 2. I choose food that is good for my health 3. I read about what is considered to be healthy food 4. I eat according to current health guidelines 5. I think about eating high quality fats, proteins and carbohydrates 6. Nutrition is more important for me than taste when it concerns food 7. Food affects my health |
|  | **The health concerned exerciser** | 1. I am most and foremost physically active to maintain my weight 2. I am most and foremost physically active to manage an existing injury or disease 3. I am most and foremost physically active to prevent a injury or disease 4. I establish goals for my training 5. I read about what is considered to be good exercise for health 6. If I am injured or sick I still try to exercise |
